# Nanostructure and Fracture Behavior of Carbon Nanofiber-Reinforced Cement Using Nanoscale Depth-Sensing Methods

**DOI:** 10.3390/ma13173837

**Published:** 2020-08-31

**Authors:** Ange-Therese Akono

**Affiliations:** 1 Department of Civil and Environmental Engineering, Northwestern University, Evanston, IL 60208, USA; ange-therese.akono@northwestern.edu; 2 Department of Mechanical Engineering, Northwestern University, Evanston, IL 60208, USA

**Keywords:** carbon nanofibers, cement, fracture toughness, calcium silicate hydrates, pore structure, nanostructure

## Abstract

In recent years, carbon nanofibers have been investigated as a suitable reinforcement for cementitious composites to yield novel multifunctional materials with improved mechanical, electrical, magnetic, and self-sensing behavior. Despite several studies, the interactions between carbon nanofibers and Portland cement hydration products are not fully understood, with significant implications for the mechanical response and the durability at the macroscopic lengthscale. Thus, the research objective is to investigate the influence of carbon nanofibers on the nanostructure and on the distribution of hydration products within Portland cement paste. Portland cement w/c = 0.44 specimens reinforced with 0.0 wt%, 0.1 wt%, and 0.5 wt% CNF by mass fraction of cement are cast using a novel synthesis procedure. A uniform dispersion of carbon nanofibers (CNF) via a multi-step approach: after pre-dispersing carbon nanofibers using ultrasonic energy, the carbon nanofibers are further dispersed using un-hydrated cement particles in high shear mixing and mechanical stirring steps. High-resolution scanning electron microscopy analysis shows that carbon nanofibers fill nanopores and connect calcium–silicate hydrates (C–S–H) grains. Grid nano-indentation testing shows that Carbon nanofibers influence the probability distribution function of the local packing density by inducing a shift towards higher values, η = 0.76–0.93. Statistical deconvolution analysis shows that carbon nanofibers result in an increase in the fraction of high-density C–S–H by 6.7% from plain cement to cement + 0.1 wt% CNF and by 10.7% from plain cement to cement + 0.5 wt% CNF. Moreover, CNF lead to an increase in the C–S–H gel porosity and a decrease in both the capillary porosity and the total porosity. Based on scratch testing, adding 0.1 wt% CNF yields a 4.5% increase in fracture toughness and adding 0.5 wt% CNF yields a 7.6% increase in fracture toughness. Finally, micromechanical modelling predicts an increase of respectively 5.97% and 21.78% in the average Young’s modulus following CNF modification at 0.1 wt% CNF and 0.5 wt% CNF levels.

## 1. Introduction

Carbon nanofiber-reinforced concrete is very attractive as a new breed of smart and multifunctional construction materials that are electrically conductive, magnetic, and self-sensing. Prior research has shown that carbon nanofibers can enhance the electrical sensitivity [1] and the strain sensing capabilities of self-consolidating concrete [2]. Moreover, carbon nanofiber concretes with 1 wt% and 2 wt% CNF reinforcement have displayed excellent strain sensing [3] and damage-sensing [4] capabilities. Consequently, carbon nanofiber concrete has been studied in structural health monitoring and in electromagnetic interference shielding applications [5,6]. Given the important role of cement paste in dictating the behavior of concrete [7], a basic understanding of the effect of CNF on cement is indispensable to design enhanced-performance and multifunctional concretes.

Carbon nanofibers (CNF) have a strong potential to enhance the mechanical behavior of cement paste [8]. Yet, a major challenge resides in achieving an optimal dispersion of carbon nanofibers within the cement matrix. The issue is the presence of strong Van der Waals forces that promote carbon nanofiber agglomeration. Yazdanbakhsh et al. [9] showed that the efficiency of the CNF dispersion procedure is function of the superplasticizer and of the cement grain size. Tyson et al. [10] cast cement nanocomposites with 0.1–0.2 wt% CNF. The CNFs were dispersed using a high-range polycarboxylate-based water reducing admixture along with ultrasonication. They reported an increase in the strength and Young’s modulus after 21 days of curing. Abu Al-rub et al. [11] investigated the influence of surface functionalization of CNF by treatment with an acidic solution on the mechanical properties of CNF nanocomposites: although the surface functionalization step increased the interfacial bonding between CNF and the cement matrix, a decrease in Young’s modulus was observed due to the formation of ettringite phases. Metaxa et al. [12,13] reported a nonlinear improvement in the flexural strength for CNF cement nanocomposites with 0.025–0.1 wt% CNF, and with 0.048 wt% CNF yielding the optimum flexural strength. Their optimum dispersion procedure involved ultrasonication of CNF in an aqueous surfactant solution with a surfactant-to-CNF weight ratio of 4.0, and with an ultrasonic energy input of 2800 kJ/L. Further studies by Gdoutos et al. [14] revealed a significant increase in the Young’s modulus and the fracture toughness for mortar with 0.1 wt% CNF. Moreover, Sanchez and coworkers [15,16] reported a secondary disaggregation of CNF following dispersion in water with and without a high-range water reducing agent. They analyzed the microstructure of CNF cement nano-composites for various dispersion procedures, using an ultrasonic bath, a sonicator probe, and using a high-range water reducing agent; in each, case they observed CNF agglomerates 7–50 μm in size present within the cement matrix. The CNF agglomerates were present even when an improvement in the flexural strength was recorded. Nevertheless, the size of the agglomerates was reduced with the use of a high-range water reducing agent. Furthermore, carbon nanofibers have been shown to yield significant improvement in the mechanical, transport, and durability properties of ultrahigh performance concrete [17,18].

Moreover, carbon nanofibers can tremendously improve the durability of cement paste. Brown and Sanchez [19] observed reduced cracking and limited changes in compressive strength in CNF cement following exposure to sulfate attack. Wang et al. [20] reported a decrease in total porosity for CNF-modified cement nanocomposites. Furthermore, Blandine et al. [21] reported a drastic reduction in autogenous shrinkage for Portland cement paste reinforced with 0.1 wt% CNF.

Despite big strides in the science of CNF-modified cements, a fundamental understanding of the interaction of carbon nanofibers with cement hydration products is still needed. The prevailing hypotheses are that carbon nanofibers accelerate the early hydration of cement, provide load transfer between cement hydration products [12], refine the pore size [19], and fill and bridge nanocracks [10] and voids [20]. Yet, additional studies are needed to understand the influence of carbon nanofibers on cement nanostructure. A recent study by Barbhuiya and Chow [22] showed that carbon nanofiber increase the fraction of high-density C–S–H in cement + 0.2 wt% CNF. The research objective in this study is to go one step further by employing advanced depth-based sensing methods such as grid nanoindentation and scratch testing along with nonlinear micromechanical modeling to elucidate the impact of carbon nanofiber modification on the distribution of cement hydration products, on the pore structure, and on the calcium silicate hydrate gel porosity of cement nanocomposites.

## 2. Materials and Methods

### 2.1. Materials Synthesis

Carbon nanofiber-modified cement nano-composites with carbon nanofibers weight fractions of 0.0 wt%, 0.1 wt%, and 0.5 wt% by mass fraction of cement and with a constant water-to-cement ratio w/c = 0.44 were synthesized as shown in Table 1. The reference material consisted of 69.44 g of type I Portland cement, and 30.56 g of deionized water. The chemical composition of the type I Portland cement utilized is provided in Table 2. The 0.1 wt% and 0.5 wt% carbon nanofiber (CNF) cement nano-composites contained in addition, respectively 0.069 g and 0.347 g, of carbon nanofibers per 100 g of material. Vapor-grown carbon nanofibers were acquired from Pyrograph Products, Inc. (Cedarville, OH, USA) [13]. The carbon nanofibers were highly graphitic and tubular with an average diameter of 150 nm, a 50–200 μm length, and a surface area of 20–30 m${}^{2}$/g.

**Figure 1:**
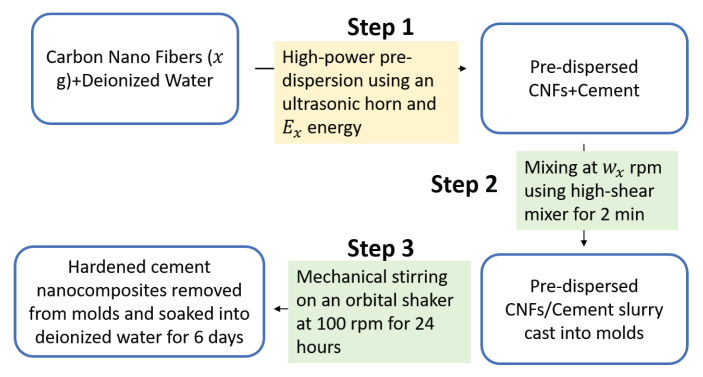
Experimental protocol employed to synthesize cement nanocomposites reinforced with carbon nanofibers.

The carbon nanofibers were dispersed in the cement matrix using first high-energy ultrasonic energy and then unhydrated cement particles under high shearing and mechanical stirring. The CNF cement nano-composites were synthesized in three steps as shown in Figure 1. First, the carbon nanofibers were pre-dispersed in deionized water using a Sonics VCX-750 ultrasonic processor (Sonics & Materials Inc, Newton, CT, USA) with a total energy output of 0 kJ, 2 kJ, and 10 kJ, corresponding respectively to the reference Portland cement paste, the 0.1 wt% CNT cement, and 0.5 wt% CNF cement specimens. Second, the suspension of CNF dispersed in deionized water was mixed with cement paste using an IKA RW 20 overhead stirrer (IKA Works Inc., Wilmington, NC, USA) equipped with a four-blade propeller for two minutes at a speed of 200 rpm for the reference specimen and 0.1 wt% CNF cement and 800 rpm for the 0.5 wt% CNF cement specimen. Afterward, the resulting slurry was poured into lubricated 32-mm round cylindrical molds. The cast cementitious specimens were initially cured for 24 h at 22 ± 2 ${}^{\circ }$C using an orbital shaker with a 3-mm radius orbit at a speed of 100 rpm. Then, the specimens were demolded and soaked in deionized water for six additional days at 22 ± 2 ${}^{\circ }$C. Finally, after seven days of curing, the cement nanocomposites were soaked in ethanol for 24 h to stop the cement hydration reaction, then wrapped in polyethylene films and stored in vacuum desiccators.

### 2.2. Methods

#### 2.2.1. Grinding and Polishing

The cement nano-composites were polished in three steps to yield a thoroughly flat surface. First, the 32-mm CNF cement specimens were embedded in low-viscosity epoxy resin and sectioned using a low-speed diamond saw to yield 5-mm thick slices. Second, after mounting on aluminum discs using cyano-acrylate adhesive, the specimens were ground using a semi-automated grinder polisher in concert with silicon carbide grinding pads of grit size 400, 600, and 800. Afterward, the specimens were manually polished using silicon carbide polishing pads of particle size 3 μm, 1 μm, and 0.25 μm. In between each step, the specimens were cleansed in N-decane using an ultrasonic bath for two minutes. At the end of the grinding and polishing procedure, the specimens were stored in vacuum desiccators.

#### 2.2.2. Scanning Electron Microscopy Imaging

In order to visualize the microstructure of the CNF cement nano-composites, environmental scanning electron microscopy imaging was performed on uncoated polished specimens. To this end, an environmental scanning electron microscope was used in backscattered mode and under low-vacuum at the Northwestern University Electron Probe Instrumentation Center. The accelerating voltage was 15 kV, the walking distance was 11 mm, and the magnification level ranged from 55× to 20,000×.

#### 2.2.3. Nanoindentation Testing

In order to map the elasto-plastic properties, nanoindentation testing was conducted using an Anton Paar nanohardness tester NHT${}^{2}$ (Anton Paar, Ashland, VA, USA). Each individual indentation test was characterized by a maximum vertical force of 2 mN, a loading/unloading rate of 4 mN/min, and a holding phase of 10 s. For each sample, a 20 × 20 array of indentation tests was conducted with an inter-indent spacing of 20 μm. For each indent, the local values of the indentation modulus, $M_{i}$ and of the indentation hardness, $H_{i}$ were measured from the load-depth curve by application of the Oliver & Pharr model [23]:

(1) $$\frac{1}{E_{r}}=\frac{1-\nu^{2}}{E}+\frac{1}{M_{i}};\;H_{i}=\frac{P_{max}}{A_{i}\left(h_{i}\right)},$$

where $E_{r}=\frac{\sqrt{\pi }}{2}\frac{S}{\sqrt{A_{i}\left(h_{i}\right)}}$ is the reduced modulus which is calculated knowing *S*, the slope of the load-depth curve upon unloading. $P_{max}=2$ mN is the maximum force, and $A_{i}$ is the projected load contact area at maximum penetration depth $h_{i}$. All tests were performed using a diamond Berkovich probe with E=1024 GPa and ν=0.17 being respectively the Young’s modulus and Poisson’s ratio of the probe. Prior to running the series of indentation tests, the curve A(h) was calibrated using fused silica as reference material.

For each individual indentation test, the local value of the packing density $\eta_{i}$ was measured by application of theoretical solutions for indentation in porous cohesive frictional materials [24]:

(2) $$\begin{array}{c} M_{i}=m_{s}M\left(\eta_{i},\nu_{s}\right);\;H_{i}=c_{s}H\left(\eta_{i},\alpha_{s},\nu_{s}\right). \end{array}$$

Herein, $m_{s}$, $\nu_{s}$, $c_{s}$, and $\alpha_{s}$ are respectively the plane strain elastic modulus, Poisson’s ratio, cohesion, and coefficient of internal friction of the solid skeleton. M is the indentation modulus linear upscaling function which is a function of the morphology as well as the packing density. For instance, for a statistically disordered morphology with spherical spheres and for $\nu_{s}=0.2$, we have M(η)=2η−1 [25]. Meanwhile, the indentation hardness nonlinear upscaling function, H, can be derived via yield design analysis [24,26].

Statistical deconvolution analysis was conducted based on the distribution ${\left(M_{i},H_{i},\eta_{i}\right)}_{1\leq i\leq 400}$ so as to identify individual chemo-mechanical phases based on their mechanical signature. The principle of statistical deconvolution methods is to represent a convoluted probability distribution function as a weighted sum of individual Gaussian functions [27,28]. In the case of cement paste nanocomposites we expect to find n=5 types of phases: capillary pores, low-density calcium silicate hydrates (C–S–H), high-density C–S–H, ultra-high density C–S–H, and unhydrated clinker [25]. Each phase *j* is characterized by five mechanical parameters: the surface fraction $f_{j}$, the mean indentation modulus and hardness, $\mu_{j}^{M}$ and $\mu_{j}^{H}$ and the standard deviations of the indentation modulus and the hardness, $s_{j}^{M}$ and $s_{j}^{H}$. The 5×n unknowns ${\left\{f_{j},\mu_{j}^{M},s_{J}^{M},\mu_{j}^{H},s_{j}^{H}\right\}}_{\left(j\in [1,n]\right)}$ are determined by minimizing the squared sum difference between the experimental probability distribution functions $PDF_{X}\left(X_{i}\right)$ and the weighted model probability distribution functions $PDF(X_{i},\mu_{j}^{X},s_{j}^{X})$ according to:

(3) $$\begin{array}{c} \min\sum_{i=1}^{N}\sum_{X=(M,H)}{\left(\sum_{j=1}^{n}f_{j}PDF\left(X_{i},\mu_{j}^{X},s_{j}^{X}\right)-PDF_{X}\left(X_{i}\right)\right)}^{2}. \end{array}$$

Moreover, we enforce two additional constraints. First, the sum of the surface fractions of each phase should be equal to unity: $\sum_{j=1}^{n}f_{j}=1$. Second, we seek to avoid a phase overlap between neighboring Gaussian curves: $\mu_{j}^{X}+s_{j}^{X}\leq \mu_{j+1}^{X}-s_{j+1}^{X}$.

#### 2.2.4. Scratch Testing

The fracture behavior was measured using microscopic scratch tests with an Anton Paar micro-scratch tester. In the scratch test experiments, a Rockwell C diamond probe was pushed across the surface of the specimen under a prescribed linearly-increasing vertical force while the horizontal force and the penetration depth were measured using high-accuracy force and displacement transducers at an acquisition rate of 45 kHz. The maximum vertical force was 2 N, the scratch length was 3 mm, and the scratch speed was 6 mm/min. Prior to testing, the surface baseline was probed using a contact force of 3 mN. The fracture toughness $K_{c}$ was computed from the horizontal force $F_{T}$ and penetration depth *d* measurements using [29,30]:

(4) $$\begin{array}{c} \frac{F_{T}}{\sqrt{2pA}}=K_{c}, \end{array}$$

2pA is the scratch probe shape function that was calibrated prior to scratch testing using fused silica as a reference material.

#### 2.2.5. Micromechanical Modeling

The elastic properties of CNF-modified cement were upscaled using a linear micromechanics scheme. At the nanoscale, each C–S–H hydration product *i* consists of C–S–H gel pores and of a C–S–H solid skeleton with a packing density $\eta_{i}$. Therefore, for each C–S–H hydration product i={LDC–S–H,HDC–S–H,UHDC–S–H}, the effective stiffness tensor is that of a porous solid of packing density $\eta_{i}$, which is given by [31,32]:

(5) $$\begin{array}{c} C_{i}=\eta_{i}C^{\left(s\right)}:A^{\left(s\right)};\;A^{\left(s\right)}={\left[\left(1-\eta_{i}\right){\left(I-P_{0}:C^{\left(s\right)}\right)}^{-1}+\eta_{i}I\right]}^{-1}, \end{array}$$

where $\eta_{i}$ is the packing density and $A^{\left(s\right)}$ is the strain concentrator tensor for the solid skeleton phase. Meanwhile, $P_{0}$ is the Hill tensor for a spherical inclusion in an elastic isotropic medium. $P_{0}$ is given by: $P_{0}=\frac{1}{3\kappa_{s}+4\mu_{s}}J+\frac{3}{5\mu_{s}}\frac{\kappa_{s}+2\mu_{s}}{3\kappa_{s}+4\mu_{s}}K$ [33]. $C^{\left(s\right)}=3\kappa_{s}J+2\mu_{s}K$ is the stiffness tensor of the C–S–H solid skeleton. I, J, and K are respectively the symmetric fourth-order unit tensor, the fourth-order spherical tensor, and the fourth-order deviatoric projection tensor. Finally, $\kappa_{s}$ and $\mu_{s}$ are respectively the bulk modulus and shear modulus of the solid skeleton.

At the submicron scale, the C–S–H matrix consists of low-density (LD) C–S–H, high-density (HD) C–S–H, and ultra-high density (UHD) C–S–H phases arranged in a statistically disordered fashion. The effective stiffness tensor of the C–S–H matrix then reads:

(6) $$\begin{array}{cc} C_{C–S–H\;\mathrm{Matrix}} & =\phi_{\mathrm{LD}\;C–S–H}C_{\mathrm{LD}\;C–S–H}:A_{\mathrm{LD}\;C–S–H}+\\ & \phi_{\mathrm{HD}\;C–S–H}C_{\mathrm{HD}\;C–S–H}:A_{\mathrm{HD}\;C–S–H}+\\ & \phi_{\mathrm{UHD}\;C–S–H}C_{\mathrm{UHD}\;C–S–H}:A_{\mathrm{UHD}\;C–S–H}. \end{array}$$

Given a phase i={LDC–S–H,HDC–S–H,UHDC–S–H}, $\phi_{i}$, $C_{i}$, and $A_{i}$ are respectively the volume fraction of phase *i* within the C–S–H matrix, the stiffness tensor of phase *i*, and the strain concentration tensor of phase *i*. The strain concentration tensor of phase *i* is given by [33]:

(7) $$\begin{array}{cc} A_{i} & =\phi_{i}\tilde{A_{i}}:{\left[\sum_{j=\{\mathrm{LD}\;C–S–H,\mathrm{HD}\;C–S–H,\mathrm{UHD}\;C–S–H\}}\phi_{j}\tilde{A_{j}}\right]}^{-1} \end{array}$$

(8) $$\begin{array}{cc} \tilde{A_{j}} & ={\left(I+P_{C–S–H\;\mathrm{Matrix}}:\left(C_{j}-C_{C–S–H\;\mathrm{Matrix}}\right)\right)}^{-1}. \end{array}$$

A self-consistent scheme was employed where the reference material is the C–S–H matrix. As a result, the Hill tensor reads: $P_{C–S–H}\;\mathrm{Matrix}=\frac{1}{3\kappa_{C–S–H}\;\mathrm{Matrix}+4\mu_{C–S–H}\;\mathrm{Matrix}}J+\frac{3}{5\mu_{C–S–H}\;\mathrm{Matrix}}\frac{\kappa_{C–S–H}\;\mathrm{Matrix}+2\mu_{C–S–H}\;\mathrm{Matrix}}{3\kappa_{C–S–H}\;\mathrm{Matrix}+4\mu_{C–S–H}\;\mathrm{Matrix}}K$. $\kappa_{C–S–H}\;\mathrm{Matrix}$ and $\mu_{C–S–H}\;\mathrm{Matrix}$ are respectively the homogenized bulk modulus and shear modulus for the C–S–H matrix.

At the microscopic length-scale, capillary pores and unreacted clinker grains are embedded within the C–S–H matrix. The effective stiffness tensor for the cement paste reads then:

(9) $$\begin{array}{cc} C_{\mathrm{Cement}\;\mathrm{Paste}} & =\phi_{C–S–H\;\mathrm{matrix}}C_{C–S–H\;\mathrm{matrix}}:A_{C–S–H\;\mathrm{matrix}}+\\ & \phi_{\mathrm{Clinker}}C_{\mathrm{Clinker}}:A_{\mathrm{Clinker}} \end{array}$$

$C_{\mathrm{Clinker}}$ is the stiffness tensor of unreacted clinker and $\phi_{\mathrm{Clinker}}$ is the volume fraction of unreacted clinker grains. $\phi_{C–S–H\;\mathrm{matrix}}$ is the volume fraction of the C–S–H matrix. We employ a Mori-Tanaka scheme to capture the matrix-inclusion morphology. The reference material is the C–S–H matrix. Therefore, the strain concentration tensors are given by:

(10) $$\begin{array}{cc} A_{C–S–H\;\mathrm{matrix}} & =\phi_{C–S–H\;\mathrm{matrix}}{\left[\phi_{C–S–H\;\mathrm{matrix}}I+\phi_{\mathrm{Clinker}}\tilde{A_{\mathrm{Clinker}}}\right]}^{-1} \end{array}$$

(11) $$\begin{array}{cc} A_{\mathrm{Clinker}} & =\phi_{\mathrm{Clinker}}\tilde{A_{\mathrm{Clinker}}}:{\left[\phi_{C–S–H\;\mathrm{matrix}}I+\phi_{\mathrm{Clinker}}\tilde{A_{\mathrm{Clinker}}}\right]}^{-1} \end{array}$$

with $\tilde{A_{\mathrm{Clinker}}}={\left(I+P_{C–S–H\;\mathrm{Matrix}}:\left(C_{\mathrm{Clinker}}-C_{C–S–H\;\mathrm{Matrix}}\right)\right)}^{-1}$. In particular, the Hill tensor is still $P_{C–S–H}\;\mathrm{Matrix}$.

## 3. Results

### 3.1. Microstructural Characteristics

**Figure 2:**
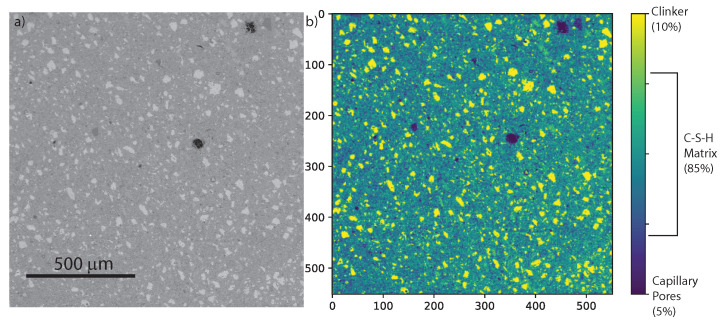
(**a**) Environmental backscattered electron microscopy (ESEM) image of CNF cement nanocomposite (0.5 wt% CNF) at a 55× magnification level. The C–S–H matrix is in dark grey, clinker grains are in light grey, and capillary pores are in black. (**b**) Digital image analysis of ESEM image of CNF-modified cement.

Figure 2a displays an environmental backscattered scanning electron microscopy image of a CNF cement nanocomposite, with 0.5 wt% CNF, at a magnification level of 55×. A porous, heterogeneous, and granular microstructure is observed. There is a dominating dark grey matrix which is the calcium–silicate hydrates (C–S–H) matrix. Unhydrated clinker grains crystals, 50 μm wide, can be seen in light grey. Finally, a few capillary pores, 50 μm wide, are shown in black. Furthermore, see Figure 2b, digital image analysis reveals that capillary pores represent 5% of the surface area, unhydrated clinker grains occupy 10% of the total surface area, whereas the C–S–H matrix occupy 85% of the total surface area.

**Figure 3:**
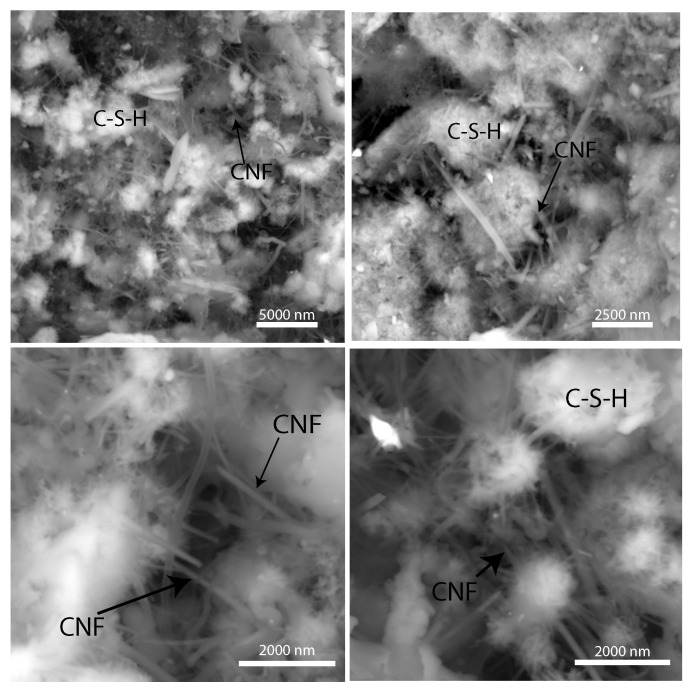
Environmental backscattered electron microscopy image of the calcium–silicate hydrate (C–S–H) matrix within the CNF cement nanocomposite (0.5 wt% CNF) at a 20,000× magnification level.

Figure 3 displays a high resolution scanning electron (SEM) image of the C–S–H matrix, with a magnification level of 20,000×. A heterogeneous, porous, and granular microstructure is observed with C–S–H grains in the size of 2500 nm. Carbon nanofiber bundles can be seen filling nanopores. Moreover, carbon nanofiber bundles also emerge from C–S–H grains suggesting that carbon nanofibers promote the nucleation of C–S–H crystals. Finally, carbon nanofiber bundles can also be seen connecting C–S–H grains, leading to bridging effect, and facilitating load transfer. Thus, the SEM experiments show that carbon nanofiber cement nanocomposites exhibit a multiscale structure and that carbon nanofibers affect the microstructure at the nanometer level.

### 3.2. Probabilistic Description of the Mechanical Behavior

Grid nanoindentation was utilized to map the elasto-plastic behavior. The local packing density was computed by application of Equation (2). The calibrated values for the intrinsic parameters of the C–S–H solid skeleton are: $\nu_{s}=0.2$, $m_{s}=63.5$ GPa, $c_{s}=0.264$ GPa, and $\alpha_{s}=0.30$. These values are in agreement with reported values of the plane strain elastic modulus $m_{s}$ and of the cohesion $c_{s}$ for calcium–silicate hydrates using nanoindentation experiments as well as molecular dynamics simulations [34,35,36]. The linear and nonlinear upscaling functions M(η) and H(η) are displayed in the supplementary document: a good agreement was observed between theory and experiments.

**Figure 4:**
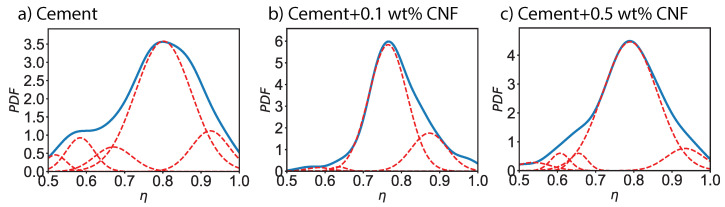
Probability distribution functions (PDF) of the local packing density (η) for plain cement and for CNF-modified cement nanocomposites.

Figure 4 shows the probability distribution of the local packing density for plain cement, 0.1 wt% CNF-, and 0.5 wt% CNF-modified cement specimens. The probability distribution function for plain cement exhibits three major peaks: η=0.58, η=0.80, and η=0.92, with the second peak being dominant. For 0.1 wt% CNF-modified cement, the first peak is significantly reduced and the intensity of the second peak is increased whereas its location is shifted to η=0.76. Similarly, the third peak is shifted to η=0.87. Finally, for 0.5 wt% CNF, the intensity of the first peak is diminished and the location is moved to η=0.61. The second peak remains at η=0.79 and both the intensity and width are increased. Finally, the third peak is shifted to η=0.93 is reduced. Thus, the addition of carbon nanofibers lead to a reduction in low-density areas, with η<0.7 and an increase in the high-density area, η=0.7−0.9. In other words, carbon nanofibers contribute to densify the cement paste.

**Figure 5:**
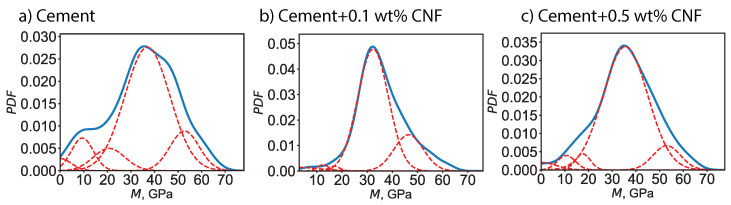
PDF of the indentation modulus (*M*) for plain cement and for CNF-modified cement nanocomposites. The dotted lines represent the probability distribution functions for individual chemo-mechanical phases, whereas the solid line the experimental collective probability distribution function based on 400 indentation tests.

**Figure 6:**
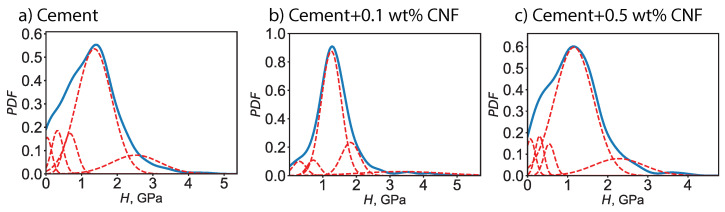
Probability distribution functions (PDF) of the indentation hardness (*H*) for plain cement and for CNF-modified cement nanocomposites. The dotted lines represent the probability distribution functions for individual chemomechanical phases whereas the solid line the experimental collective probability distribution function based on 400 indentation tests.

Moreover, a probabilistic framework was adopted to describe the elasto-plastic characteristics. For a given phase, the indentation modulus—respectively the indentation hardness—spans a continuous range values and the likelihood of each value is described by a probability distribution function. We assume a Gaussian distribution of the indentation modulus—respectively the indentation hardness—characterized by an average value and a standard deviation. The average value points to the location of the maximum whereas the standard deviation points to the half-width of the Gaussian bell curve. Figure 5 displays the computed probability distribution functions at both the collective and individual level for the indentation modulus *M*, for plain cement and for CNF-modified cement. Meanwhile, the probability distribution functions for the indentation hardness *H* are displayed in Figure 6. By using grid nanoindentation we can quantify the uncertainty on the mechanical response. The probability distribution function for the indentation modulus exhibits a main peak in the 30–40 GPa range, with the exact location of the peak and the half-bandwidth of the peak being dependent on the CNF content. Similarly, the probability distribution function for the indentation hardness exhibits a main peak in the 1–2 GPa range, with the exact location of the peak and the half-bandwidth of the peak being dependent on the CNF content.

A statistical deconvolution analysis of the indentation data was conducted to identify the cement microconstituents. Five phases are expected: capillary pores, low-density C–S–H, high-density C–S–H, ultra-high-density C–S–H, and un-hydrated clinker grains. Low-density C–S–H and high-density C–S–H are compositionally similar and structurally distinct phases of the C–S–H matrix. We use as a reference Jenning’s colloidal model for calcium silicate hydrates [37,38,39] where the C–S–H gel is a gelled colloid and a nanogranular material with the globule, 2–4 nm in size, being the basic unit. High-density C–S–H consists of C–S–H globules packed in a hexagonally-closed packed arrangement with a packing density of 0.74 and with a pore size in the range of 1.2 nm. Low-density C–S–H consists of C–S–H packed loosely with a packing density of 0.64. Moreover, on the scale of 100 nm, low-density C–S–H exhibits mesopores in the range 5–12 nm. In addition, the specific surface area of low-density C–S–H is higher than that of high-density C–S–H, whereas high-density C–S–H is more dimensionally stable. Thus, low-density C–S–H and high-density C–S–H differ in terms of packing order, packing density values, and pore size ranges [37,38,39]. The characteristics of each chemo-mechanical phase can then be quantified: volume fraction, indentation modulus, and indentation hardness, as shown in Table 3.

The statistical deconvolution approach confirms the intrinsic nature of the mechanical characteristics for low-density C–S–H and high-density C–S–H. As shown in Table 3, for low-density C–S–H, the average indentation modulus lies in the range 16.89–20.74 GPa and the average indentation hardness lies in the range 0.49–0.73 GPa. For high-density C–S–H, the average indentation modulus lies in the range 32.37–36.98 GPa and the average indentation hardness lies in the range 1.14–1.36 GPa. These computed values agree with reported values of the indentation modulus and indentation hardness for both low-density C–S–H and high-density C–S–H that had been measured using nanoindentation experiments [40]. As for ultrahigh-density C–S–H (UHD CSH), the average indentation modulus is in the range 46.50–52.74 GPa and the average indentation hardness lies in the range 2.11–2.57 GPa. Ultrahigh-density C–S–H is usually considered to be a mix of C–S–H with portlandite [40]. Finally, capillary pores exhibit very low values of both the indentation hardness and modulus with a high variability. Meanwhile, clinker phases are characterized by an indentation modulus greater than 65 GPa.

### 3.3. Influence of CNF Content on Cement Chemo-Mechanical Phases

**Figure 7:**
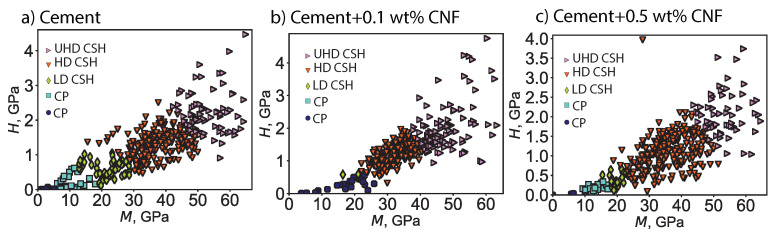
Statistical deconvolution of indentation data for plain cement and for CNF-modified cement nanocomposites. CP = capillary pores. LD CSH = low-density C–S–H. HD CSH = high-density C–S–H. UHD = ultra-high-density C–S–H. *M* is the indentation modulus and *H* is the indentation hardness. There were 400 indentation tests conducted per specimen.

**Figure 8:**
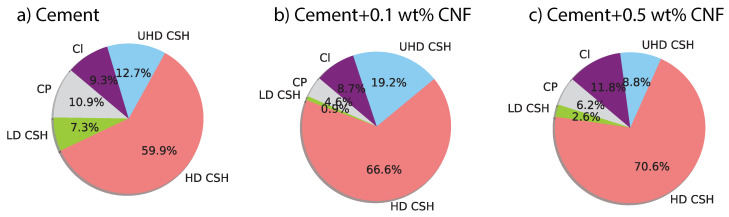
Phase distribution in both plain cement and CNF-modified cement nanocomposites. CP = capillary pores. LD CSH = low-density C–S–H. HD CSH = high-density C–S–H. UHD = ultra-high-density C–S–H. Cl = unhydrated clinker.

The addition of carbon nanofibers influences the distribution of C–S–H phases. Figure 7 displays the phase clusters for plain cement, 0.1 wt% CNF-, and 0.5 wt% CNF-modified cement. Figure 8 displays the fraction of different chemomechanical phases: capillary pores, low-density C–S–H, high-density C–S–H, ultrahigh-density C–S–H, and unhydrated clinker. Carbon nanofibers result in an increase in the fraction of high-density C–S–H by 6.7% from plain cement to cement + 0.1 wt% CNF and by 10.7% from plain cement to cement + 0.5 wt% CNF. This increase in the fraction of high-density C–S–H is positively correlated with the carbon nanofibers content in a nonlinear fashion. Moreover, the increase in high-density C–S–H is followed by a decrease in the volume fraction of low-density C–S–H by 6.4% and 5.1% respectively for cement + 0.1 wt% CNF and cement + 0.5 wt% CNF. As for ultrahigh-density C–S–H, its volume fraction reaches its maximum value for low-volume fractions of CNF, cement + 0.1 wt% CNF. Meanwhile, the volume fraction of unhydrated clinker is maximum for cement + 0.5 wt% CNF. In brief, carbon nanofibers promote the growth of high-density C–S–H.

The results from the statistical deconvolution of indentation data bring new insights regarding the influence of carbon nanofibers on the pore structure. A decrease in the fraction of capillary pores is observed, by 6.3% and 4.7% respectively for cement + 0.1 wt% CNF and cement + 0.5 wt% CNF. The C–S–H gel porosity can be computed as $\phi_{\mathrm{LD}\;\mathrm{CSH}}\left(1-\eta_{\mathrm{LD}\;\mathrm{CSH}}\right)+\phi_{\mathrm{HD}\;\mathrm{CSH}}\left(1-\eta_{\mathrm{HD}\;\mathrm{CSH}}\right)+\phi_{\mathrm{UHD}\;\mathrm{CSH}}\left(1-\eta_{\mathrm{UHD}\;\mathrm{CSH}}\right)$ where $\phi_{i,i=\{\mathrm{LD}\;\mathrm{CSH},\;\mathrm{HD}\;\mathrm{CSH},\;\mathrm{UHD}\;\mathrm{CSH}\}}$ is the volume fraction and $\eta_{i,i=\{\mathrm{LD}\;\mathrm{CSH},\;\mathrm{HD}\;\mathrm{CSH},\;\mathrm{UHD}\;\mathrm{CSH}\}}$ is the packing density of low-density C–S–H, high-density C–S–H, and ultrahigh-density C–S–H. The computed C–S–H gel porosity for plain cement, cement + 0.1 wt% CNF and cement + 0.5 wt% CNF is respectively 15.39%, 18.80%, and 16.35%. Thus, there is a 3.41% and a 0.96% increase in C–S–H gel porosity with the addition of respectively 0.1 wt% CNF and 0.5 wt% CNF. However, for CNF-modified cement, the C–S–H gel porosity is distributed primarily among gel pores, 1.2 nm large with a significant reduction in gel mesopores, 5–12 nm large, due to the decrease of low-density C–S–H phases. The total porosity is then the sum of the capillary porosity and the C–S–H gel porosity. The computed total porosity is 26.28% for plain cement, 23.36% for cement + 0.1 wt% CNF and 22.53% for cement + 0.5 wt% CNF. Thus, there is a 2.92% and a 3.75% reduction in total porosity following addition of respectively 0.1 wt% and 0.5 wt% CNF. Thus, CNF-modification of cement paste result in a reduction of the total porosity, in a reduction of the capillary porosity, and in an increase in the fraction of small C–S–H gel pores.

### 3.4. Influence of CNF Content on Fracture Resistance

**Figure 9:**
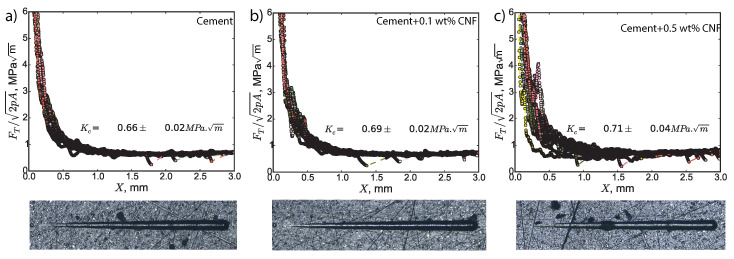
Fracture response of CNF cement nanocomposites. $F_{T}$ is the horizontal force, *X* is the scratch path, and 2pA is the scratch probe shape function. N=12 tests were performed per specimen.

The fracture response of CNF cement nanocomposites was probed using scratch tests jointly with high-resolution scanning electron microscopy imaging. As shown in Figure 9, after scratch testing, we observe the formation of a residual groove of increasing width; this residual groove points to material removal processes at work during the scratch test. For all specimens and all tests, the quantity $F_{T}/\sqrt{2pA}$ decreases and then converges toward a stable value for large scratch *X* values and for large penetration depths *d* values. For low penetration depths, the mechanical energy is dissipated through a mix a ductile and brittle processes. However, as the axi-symmetric scratch probe digs deeper into the material, the mechanical energy is dissipated primarily through brittle crack propagation processes. Thus, the convergence of $F_{T}/\sqrt{2pA}$ reflects a ductile-to-brittle transition which is activated by the penetration depth. In the asymptotic regime of a brittle regime, the asymptotic value of $F_{T}\sqrt{2pA}$ yields the fracture toughness, following Equation (1).

The presence of carbon nanofibers enhances the fracture properties of cement paste. The fracture toughness of plain Portland cement is 0.66 ± 0.02 MPa$\sqrt{m}$, value which is in agreement with macroscopic values of the fracture toughness reported in the scientific literature for plain Portland cement [41,42]. These macroscopic fracture toughness values for plain Portland cement were obtained using three-point bending tests on single-edge notched specimens in concert with large specimen extrapolation methods [41,42]. The fracture toughness of 0.1 wt% CNF cement is 0.69 ± 0.02 MPa$\sqrt{m}$ and that of 0.5 wt% CNF cement is 0.71 ± 0.04 MPa$\sqrt{m}$. Consequently, adding 0.1 wt% CNF yields a 4.5% increase in fracture toughness and adding 0.5 wt% CNF yields a 7.6% increase in fracture toughness. Thus, carbon nanofibers lead to significant gains in the fracture toughness.

### 3.5. Influence of CNF Content on Fracture Micromechanisms

**Figure 10:**
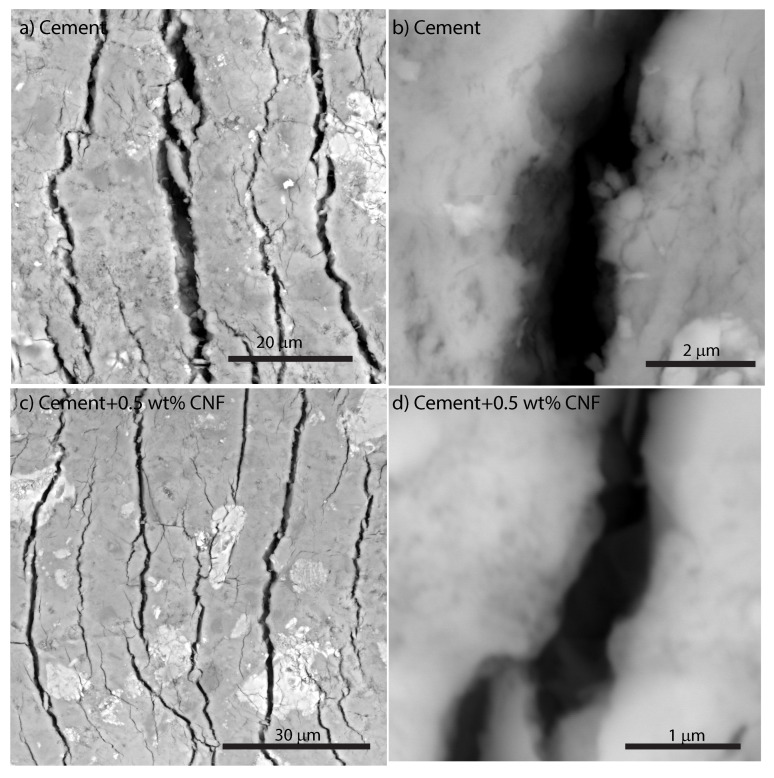
Fracture micromechanisms of 5wt% CNF cement nanocomposite compared to plain Portland cement.

In order to understand the reason for the enhancement in fracture behavior in CNF-modified cement nanocomposites, the relevant fracture micromechanisms were investigated using backscattered electron microscopy as shown in Figure 10. Figure 10a displays the residual groove of plain cement from the top: curved fracture surfaces regularly spaced can be seen. These fracture surfaces provide additional physical evidence of fracture processes during scratch testing. In addition, some common toughening mechanisms for Portland cement are observed such as: microcracking, crack deflection, ligament bridging, and particle bridging. Figure 10b presents a close-up view of a fracture surface; the crack opening averages 2 μm in width. In Figure 10c similar fracture micromechanisms are observed for 0.5 wt% CNF cement with a major difference: the fracture openings are smaller. Figure 10d provides a high-resolution imaging of the fracture surface for 0.5 wt% CNF cement: the crack opening averages 1 μm in width. Thus, the addition of carbon nanofibers results in a drastic reduction in the crack width in CNF-modified cement nanocomposites.

## 4. Discussion

### 4.1. Multiscale Conceptual Model for CNF-Modified Cement

**Figure 11:**
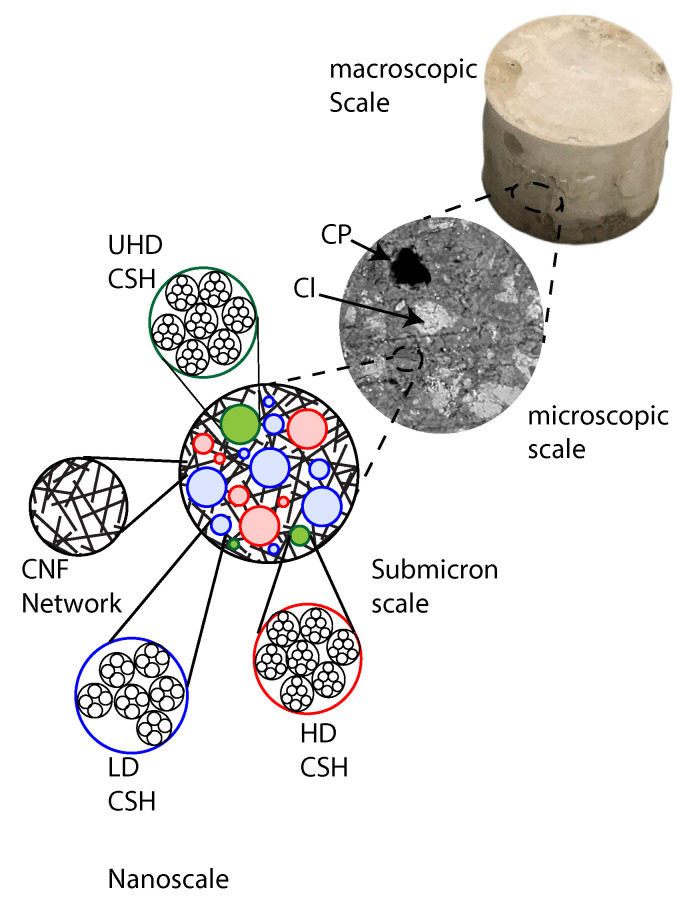
Multiscale thought-model of CNF-modified cement nanocomposite. CP = capillary pores. LD CSH = low-density C–S–H. HD CSH = high-density C–S–H. UHD = ultra-high-density C–S–H. Cl = unhydrated clinker.

A multiscale thought-model for carbon nanofiber-modified cement was formulated based on scanning electron microscopy observations. Figure 11 displays the proposed conceptual model. At the nanoscale, calcium silicate hydrate products are packed in various structural arrangements resulting in low-density C–S–H, high-density C–S–H, and ultrahigh-density C–S–H. At the submicron length-scale, C–S–H grains are connected by a network of carbon nanofibers forming the C–S–H matrix. At the microscopic length-scale, capillary pores and unhydrated clinker grains are embedded in the C–S–H matrix.

**Figure 12:**
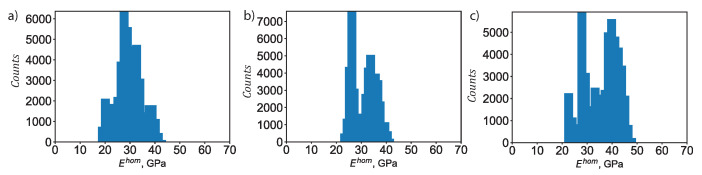
Histogram of the predicted macroscopic Young’s modulus, $E^{hom}$. (**a**) Plain Portland cement. (**b**) Cement + 0.1 wt% CNF. (**c**) Cement + 0.5 wt% CNF. For each specimen, 50,625 numerical simulations were conducted.

Through the micromechanics scheme, the elastic properties at the macroscopic scale can be computed based on the nanoindentation measurements using Equations (5)–(11). A Monte Carlo approach was employed, where we account for the stochastic variation of the clinker phase and of the packing density within the C–S–H phases—low-density C–S–H, high-density C–S–H, and ultrahigh-density C–S–H. For each specimen, we conducted 50,625 numerical simulations. Figure 12 displays the histogram of the predicted macroscopic Young’s modulus $E^{hom}$ for all three specimens, plain Portland cement, cement + 0.1 wt% CNF, and cement + 0.5 wt% CNF. For plain Portland cement, the histogram of the predicted macroscopic Young’s modulus exhibits a major peak at 29 GPa, and at 36 GPa. For cement + 0.1 wt% CNF, we observe two peaks: at 24 GPa, and at 35 GPa. Finally, for cement + 0.5 wt% CNF, we observe three peaks: at 23 GPa, at 29 GPa, and at 40 GPa. Thus, the presence of carbon nanofibers influences the stochastic distribution of the macroscopic Young’s modulus by inducing a shift towards higher values. Moreover, the average value of the predicted macroscopic Young’s modulus is 29.66 GPa, 31.43 GPa, and 36.12 GPa, respectively for plain Portland cement, cement + 0.1 wt% CNF, and cement + 0.5 wt% CNF.

Thus, the micromechanics analysis predicts an increase of respectively 5.97% and 21.78% in the average Young’s modulus following CNF modification at 0.1 wt% CNF and 0.5 wt% CNF levels. Using flexural tests, Abu Al-rub et al. [11] also reported an increase in the Young’s modification following nanoscale modification of Portland cement w/c = 0.4 with carbon nanofibers. However, they noted an increase in the Young’s modulus after 28 days of curing. Furthermore, they used a superplasticizer agent along with sonication to disperse the nanomaterials. Metaxa et al. [13] also reported an increase in the Young’s modulus for Portland cement w/c = 0.5 reinforced with 0.048 wt% CNF after both 7 days and 28 days of curing. This study is able to quantify the uncertainty in the values of the macroscopic Young’s modulus. In addition, this study ties the increase in the Young’s modulus to CNF-induced microstructural and compositional changes, specifically to the reduction in capillary pores and the increase in the volume fraction of high-density C–S–H.

### 4.2. Influence of CNF on Nanostructure

The presence of carbon nanofibers modifies the microstructure and nanostructure of CNF-modified cement. A reduction in the capillary porosity and a reduction in total porosity are observed, followed by a refinement of the pore structure. The inverse micromechanical analysis yields a shift from micropores and mesopores—100 nm large—in plain Portland cement to small gel pores, 5–12 nm large, in CNF-modified cement. These findings agree with the results of Wang et al. [20]; using ${}^{1}$H low-field nuclear magnetic resonance, they recorded a drastic reduction in porosity between plain Portland cement w/c = 0.35 and CNF-modified cement with 0.1 wt% and 0.2 wt% CNF. These findings agree also with the results of Brown and Sanchez [19] who, using Nitrogen adsorption tests, recorded a significant decrease in the fraction of mesopores—100 nm large—in cement + 0.5 wt% CNF compared to plain Portland cement w/c = 0.28. This work goes one step further, as the micromechanics-based approach can directly tie the pore refinement to an improvement in the effective mechanical response.

Furthermore, the increase in high-density C–S–H and the pore refinement observed in CNF-modified cement can explain the decrease in autogenous shrinkage reported for CNF-modified cement. Blandine et al. [21] reported a decrease in the autogenous shrinkage of Portland cement following modification with carbon nanotubes and carbon nanofibers. Within the framework of the colloidal model of C–S–H [38], shrinkage results from the irreversible motion of C–S–H globules under drying stresses as the relative humidity is lower due to the ongoing cement hydration reaction. Among the C–S–H phase, the low-density C–S–H is most susceptible to experience high irreversible volume changes given its low packing density as well as the presence of large gel mesopores. In contrast, high-density C–S–H is more dimensionally stable due to its high packing density along with a structural arrangement in close-packed hexagonally packed structures. Therefore, as the presence of carbon nanofibers promotes the formation of high-density C–S–H, carbon nanofibers will lead to a reduction in autogenous shrinkage for the cement paste. Thus, these results link the decrease in autogenous shrinkage in CNF-modified cement paste to the CNF-induced changes in nanostructure.

### 4.3. Toughening Behavior of CNF-Modified Cement

Using microscopic scratch tests, an increase in fracture toughness for CNF-modified Portland cement is noted, along with a reduction in the crack width. Similarly, Gdoutos et al. reported an increase in fracture toughness after conducting three-point bending tests on single-edge notched specimens for both cement-based mortar and cement-based mortar + 0.1 wt% CNF. Tyson et al. [10] reported an increase in the flexural toughness for cement with 0.1 wt% CNF after conducting three-point bending tests. Carbon nanofibers toughen the cement matrix by bridging cracks [10] and pores [12]. Moreover, carbon nanofibers toughen the cement matrix by reducing the capillary porosity and promoting a refinement of the pore size. The refinement of the pore size, due to an increase in high-density C–S–H, will likely increase the fractality of the fracture surface, making it harder for fractures to propagate.

### 4.4. Dispersion of CNF

In this study, carbon nanofibers are dispersed using a multi-step process that involves high-energy pre-dispersion using ultrasonic energy followed by low-energy dispersion using un-hydrated cement particles via high shear mixing and mechanical stirring. Recent work by Rocha and coworkers [43,44] has shown mentioned, please confirm that unhydrated cement particles provide an efficient means to disperse carbon nanofibers in cement matrices. In addition, a high-shear mixing step is incorporated to accelerate calcium silicate hydrate formation [45]. Finally, the mechanical stirring steps aim to continuously re-disperse carbon nanofibers, avoid secondary carbon nanofiber agglomeration [15,46], and remove microscopic air voids. This mixing procedure yields high values of the macroscopic Young’s modulus: 29.66 GPa and 36.12 GPa respectively for plain Portland cement and for cement + 0.5 wt% CNF with a w/c = 0.44 after only seven days of curing.

## 5. Conclusions

A novel synthesis procedure for carbon nanofiber-modified cement is reported: after pre-dispersing carbon nanofibers using ultrasonic energy, the carbon nanofibers are further dispersed using un-hydrated cement particles in high shear mixing and mechanical stirring steps. The influence of carbon nanofibers on the nanostructure of CNF-reinforced Portland cement after seven days of curing is further investigated using statistical deconvolution analysis, nanoindentation testing, microscopic scratch testing, and micromechanical modeling. Below are the major findings:

- The ESEM analysis shows carbon nanofiber bundles filling nanopores. Moreover, carbon nanofiber bundles also emerge from C–S–H grains, suggesting that carbon nanofibers promote the nucleation of C–S–H crystals. Finally, carbon nanofiber bundles can also be seen connecting C–S–H grains, leading to a bridging effect, and facilitating load transfer.
- For cement + 0.1 wt% CNF and cement + 0.5wt% CNF, after seven days of curing, we observe a shift of the histogram of the local packing density towards the high-density area, η = 0.7–0.9.
- Carbon nanofibers result in an increase in the fraction of high-density C–S–H: for instance, after seven days of curing, the fraction of C–S–H is increased by 6.7% from plain cement to cement + 0.1 wt% CNF and by 10.7% from plain cement to cement + 0.5 wt% CNF. Moreover, the increase in high-density C–S–H is followed by a decrease in the volume fraction of low-density C–S–H by 6.4% and 5.1% respectively for cement + 0.1 wt% CNF and cement + 0.5 wt% CNF.
- A decrease in the fraction of capillary pores is observed by 6.3% and 4.7% respectively for cement + 0.1 wt% CNF and cement + 0.5 wt% CNF.
- The computed C–S–H gel porosity for plain cement, cement + 0.1 wt% CNF and cement + 0.5 wt% CNF is respectively 15.39%, 18.80%, and 16.35%, after 7 days of curing. The computed total porosity is 26.28% for plain cement, 23.36% for cement + 0.1 wt% CNF and 22.53% for cement + 0.5 wt% CNF. Thus, CNF-modification of cement paste result in a reduction of the total porosity, in a reduction of the capillary porosity, and in an increase in the fraction of small C–S–H gel pores (1.2–2 nm in diameter).
- Adding 0.1 wt% CNF yields a 4.5% increase in fracture toughness and adding 0.5 wt% CNF yields a 7.6% increase in fracture toughness: the fracture toughness of plain Portland cement is 0.66 ± 0.02 MPa$\sqrt{m}$; the fracture toughness of 0.1 wt% CNF cement is 0.69 ± 0.02 MPa$\sqrt{m}$ and that of 0.5 wt% CNF cement is 0.71 ± 0.04 MPa$\sqrt{m}$.
- The use of carbon nanofibers result in a drastic reduction in the crack width in CNF-modified cement nanocomposites.
- A four-level multiscale micromechanical model for CNF-cement predicts an increase of respectively 5.97% and 21.78% in the average Young’s modulus following CNF modification at 0.1 wt% CNF and 0.5 wt% CNF levels. This increase in mechanical performance is due to CNF-induced compositional and microstructural changes at both the micrometer and nanometer length-scale.

In future studies, we will study the influence of carbon nanofibers on the nanostructure and mechanical properties for different values of the water-to-cement ratio and for different curing ages.

## Figures and Tables

**Table 1:** Mix design of cement reinforced with carbon nanofibers (CNFs). DIW = Deionized Water.

Specimen	Cement	Cement + 0.1 wt% CNF	Cement + 0.5 wt% CNF
CNF, wt%	0.0	0.1	0.5
CNF, g	0.000	0.069	0.347
Cement, g	69.44	69.44	69.44
DIW, g	30.56	30.56	30.56

**Table 2:** Chemical composition of type I Portland cement used in this study.

	wt%
Alite Monoclinic $Ca_{3}SiO_{5}$ (C${}_{3}$S)	73.80
Tricalcium Aluminate $Ca_{3}Al_{2}O_{6}$	12.10
Belite $Ca_{2}SiO_{4}$ (C${}_{2}$S)	9.80
Brownmillerite $Ca_{2}FeAlO_{5}$	4.30

**Table 3:** Computed physical characteristics of chemo-mechanical phases in plain cement and in CNF-modified cement. CP = capillary porosity. LD C–S–H = low-density C–S–H. HD C–S–H = high-density C–S–H. UHD C–S–H = ultrahigh-density C–S–H. For each phase, $\mu_{M}$ (respectively $\mu_{H}$) is the average value of the indentation modulus (respectively indentation hardness); whereas $\sigma_{M}$ (respectively $\sigma_{H}$) is the standard deviation of the indentation modulus (respectively indentation hardness).

	Vol (%)	($\mu_{M}$, $\sigma_{M}$), GPa	($\mu_{H}$, $\sigma_{H}$), GPa	η
**Plain Cement**				
CP	2.72	(0.00,4.73)	(0.02,0.10)	(0.52,0.03)
CP	8.17	(9.39,4.66)	(0.29,0.17)	(0.58,0.04)
LD C–S–H	7.26	(20.74,6.69)	(0.67,0.19)	(0.67,0.05)
HD C–S–H	59.89	(36.98,9.55)	(1.36,0.49)	(0.80,0.07)
UHD C–S–H	12.71	(52.74,6.20)	(2.57,0.72)	(0.92,0.05)
Clinker	9.25	(97.23,35.34)	(4.93,3.31)	N. A.
**Cement + 0.1 wt% CNF**				
CP	3.65	(0.00,10.27)	(0.26,0.17)	(0.05,0.50)
CP	0.91	(12.49,2.23)	(0.53,0.10)	(0.58,0.03)
LD C–S–H	0.91	(16.89,2.17)	(0.73,0.10)	(0.64,0.02)
HD C–S–H	66.61	(32.37,6.09)	(1.26,0.34)	(0.76,0.05)
UHD C–S–H	19.16	(46.50,5.78)	(2.11,0.51)	(0.87,0.05)
Clinker	8.75	(91.40,21.46)	(4.25,2.41)	N. A.
**Cement + 0.5 wt% CNF**				
CP	2.65	(0.00,6.32)	(0.07,0.10)	(0.54,0.05)
CP	3.53	(10.65,3.79)	(0.27,0.10)	(0.61,0.02)
LD C–S–H	2.65	(17.60,3.16)	(0.49,0.12)	(0.66,0.02)
HD C–S–H	70.60	(35.58,9.43)	(1.14,0.53)	(0.79,0.07)
UHD C–S–H	8.83	(53.00,5.76)	(2.44,0.77)	(0.93,0.05)
Clinker	11.75	(87.65,24.75)	(4.04,3.43)	N. A.

## Supplementary Materials

The following are available online at https://www.mdpi.com/1996-1944/13/17/3837/s1, Figure S1: Environmental scanning electron images of cement reinforced with 0.5% wt Carbon NanoFibers, Figure S2: Fracture micromechanisms of plain Portland cement, Figure S3: Fracture micromechanisms of Portland cement reinforced with 0.5% wt CNF, Figure S4: Upscaling linear functions for the indentation modulus M. η is the local packing density. (a) Plain Portland cement. (b) Portland cement +0.1 wt% CNF. (c) Portland cement + 0.5 wt% CNF, Figure S5: Nonlinear upscaling for the indentation hardness H. η is the local packing density. (a) Plain Portland cement. (b) Portland cement + 0.1 wt% CNF. (c) Portland cement+0.5 wt% CNF.
