# Supplementary material for: Nanostructure and Fracture Behavior of Carbon Nanofiber-Reinforced Cement Using Nanoscale Depth-Sensing Methods
Source: Materials (Basel). 2020 Aug 31;13(17):3837. doi: 10.3390/ma13173837 (PMC7504507; doi:10.3390/ma13173837)
Supplement: Supplementary file 1 [file materials-13-03837-s001.pdf]

materials

Supplementary Materials: Nanostructure and Fracture Behavior of Carbon NanoFiber-Reinforced Cement Using Nanoscale Depth-Sensing Methods

Ange-Therese Akono [1,2]

[1] Department of Civil and Environmental Engineering, Northwestern University, Evanston, 60208 IL, USA; ange-therese.akono@northwestern.edu
[2] Department of Mechanical Engineering, Northwestern University, Evanston, 60208 IL, USA

1. Scanning Electron Microscopy Images

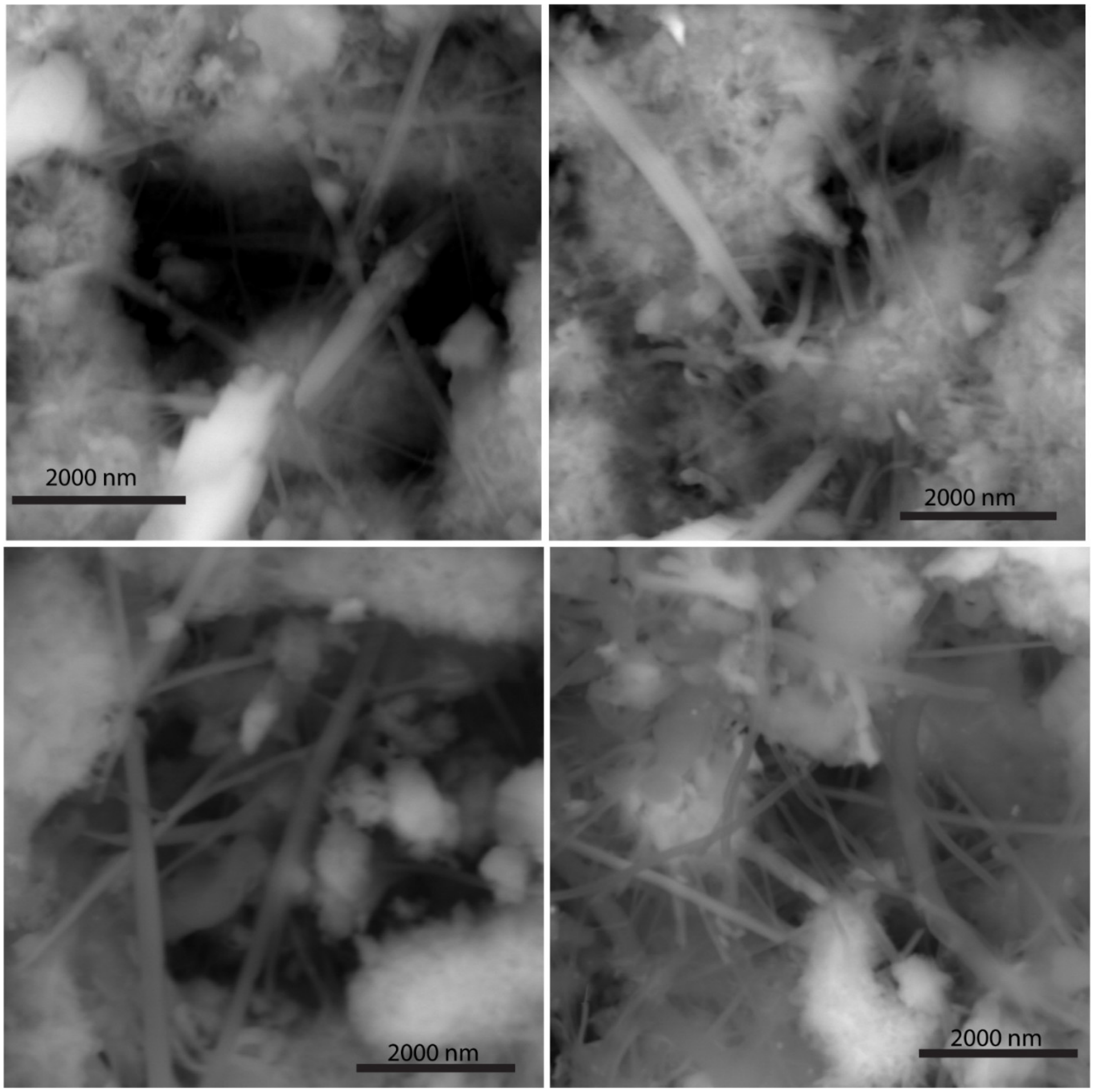

Figure S1. Environmental scanning electron images of cement reinforced with 0.5% wt Carbon NanoFibers.

2. Fractography Analyses

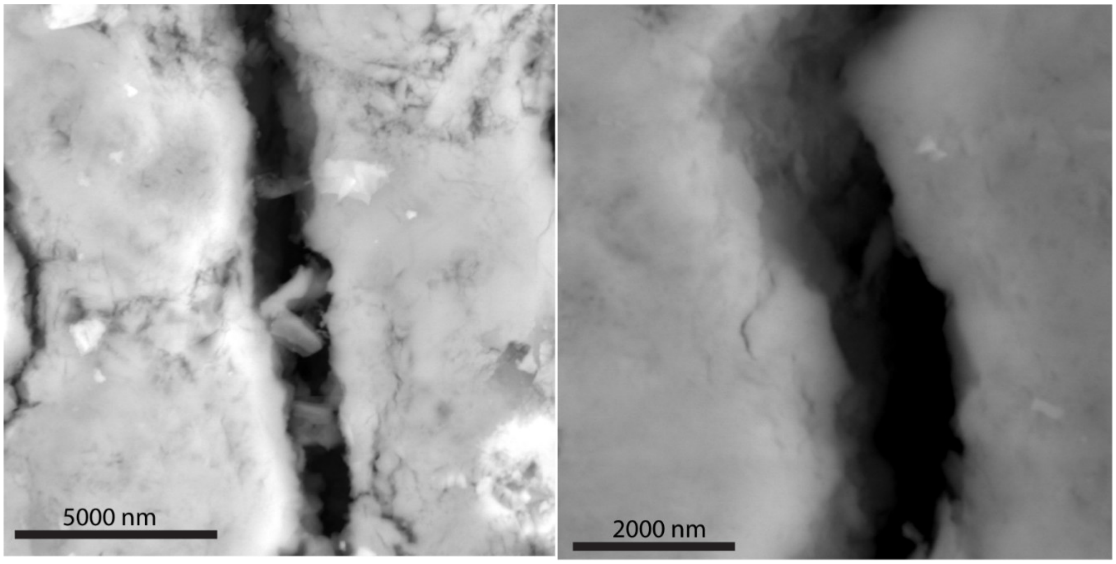

Figure S2. Fracture micromechanisms of plain Portland cement.

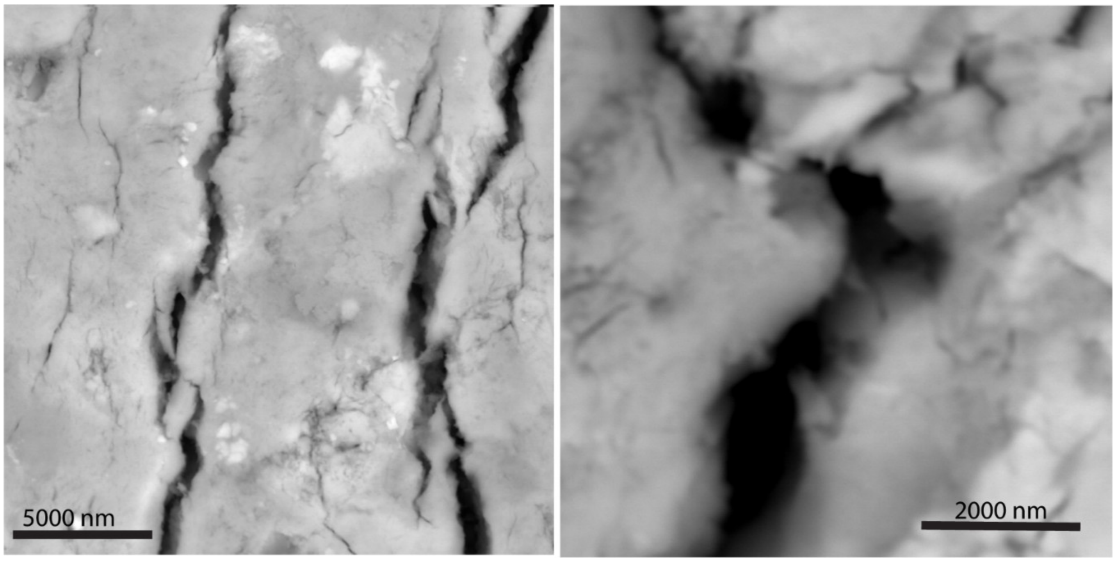

Figure S3. Fracture micromechanisms of Portland cement reinforced with 0.5% wt CNF.

3. Micromechanics Upscaling Functions

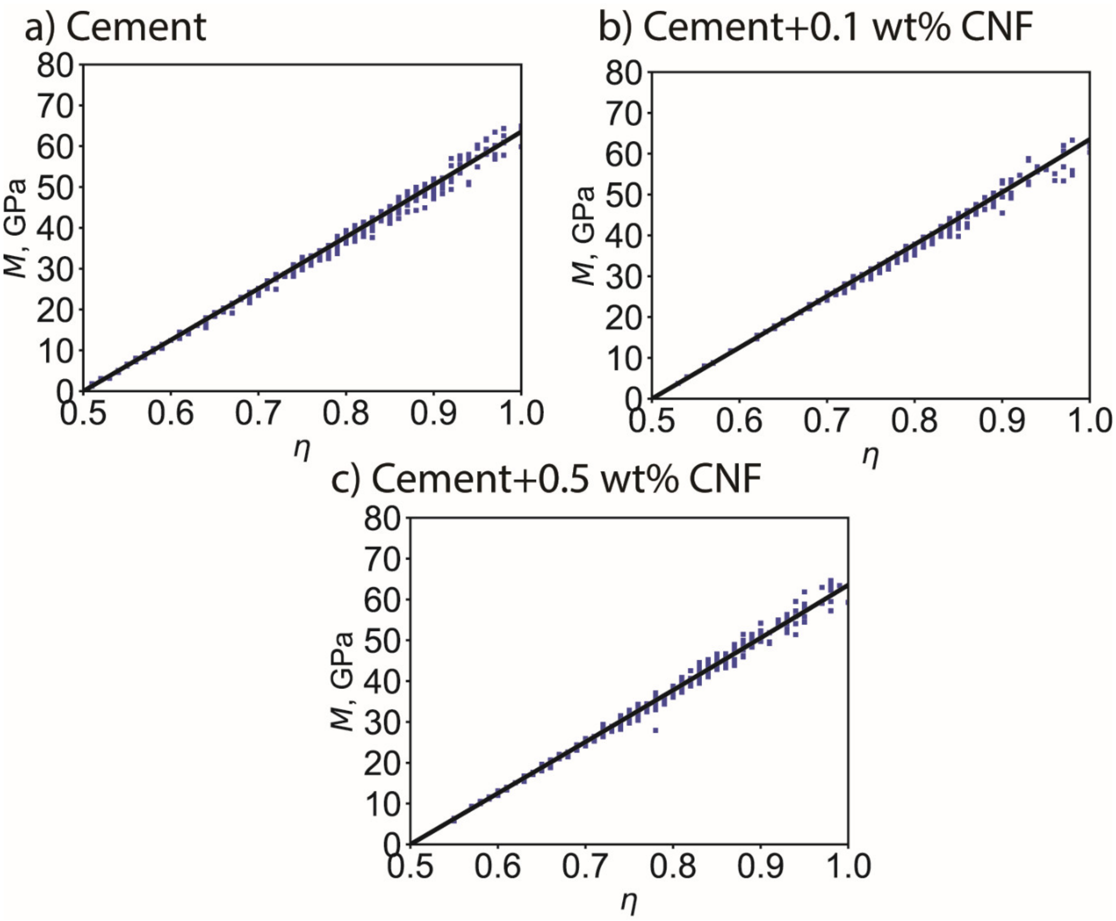

Figure S4. Upscaling linear functions for the indentation modulus M. η is the local packing density. (**a**) Plain Portland cement. (**b**) Portland cement + 0.1 wt% CNF. (**c**) Portland cement + 0.5 wt% CNF.

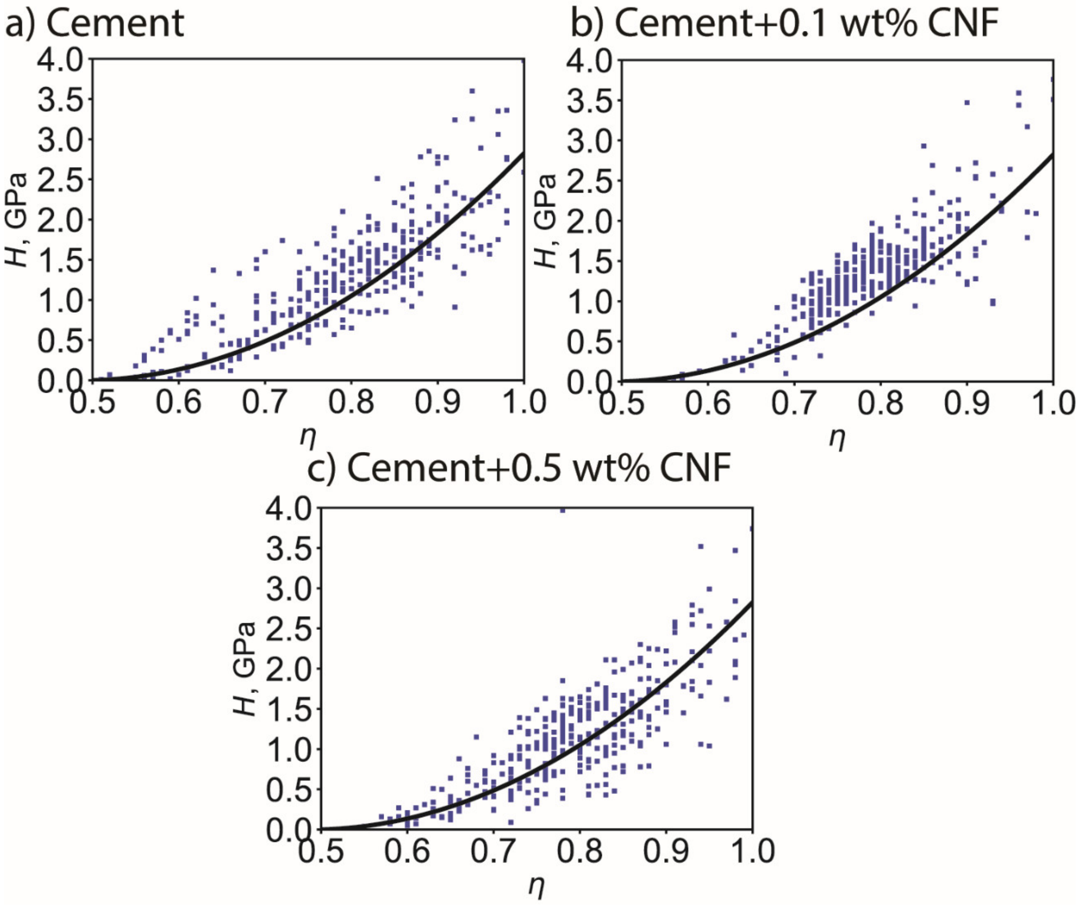

Figure S5. Nonlinear upscaling for the indentation hardness H. η is the local packing density. (**a**) Plain Portland cement. (**b**) Portland cement + 0.1 wt% CNF. (**c**) Portland cement + 0.5 wt% CNF.

© 2020 by the authors. Licensee MDPI, Basel, Switzerland. This article is an open access article distributed under the terms and conditions of the Creative Commons Attribution (CC BY) license (http://creativecommons.org/licenses/by/4.0/).
